# A Rapid Change in Virulence Gene Expression during the Transition from the Intestinal Lumen into Tissue Promotes Systemic Dissemination of *Salmonella*

**DOI:** 10.1371/journal.ppat.1001060

**Published:** 2010-08-19

**Authors:** Sebastian E. Winter, Maria G. Winter, Ivan Godinez, Hee-Jeong Yang, Holger Rüssmann, Helene L. Andrews-Polymenis, Andreas J. Bäumler

**Affiliations:** 1 Department of Medical Microbiology and Immunology, School of Medicine, University of California at Davis, Davis, California, United States of America; 2 Max von Pettenkofer-Institut für Hygiene und Medizinische Mikrobiologie, Ludwig-Maximilians-Universität München, München, Germany; 3 Department of Microbial and Molecular Pathogenesis, College of Medicine, Texas A&M University System Health Science Center, College Station, Texas, United States of America; 4 HELIOS Klinikum Emil von Behring, Institut für Mikrobiologie, Immunologie und Laboratoriumsmedizin, Berlin, Germany; University of California San Diego and Veterans Affairs (VA) Healthcare San Diego, United States of America

## Abstract

Bacterial pathogens causing systemic disease commonly evolve from organisms associated with localized infections but differ from their close relatives in their ability to overcome mucosal barriers by mechanisms that remain incompletely understood. Here we investigated whether acquisition of a regulatory gene, *tviA*, contributed to the ability of *Salmonella enterica* serotype Typhi to disseminate from the intestine to systemic sites of infection during typhoid fever. To study the consequences of acquiring a new regulator by horizontal gene transfer, *tviA* was introduced into the chromosome of *S. enterica* serotype Typhimurium, a closely related pathogen causing a localized gastrointestinal infection in immunocompetent individuals. TviA repressed expression of flagellin, a pathogen associated molecular pattern (PAMP), when bacteria were grown at osmotic conditions encountered in tissue, but not at higher osmolarity present in the intestinal lumen. TviA-mediated flagellin repression enabled bacteria to evade sentinel functions of human model epithelia and resulted in increased bacterial dissemination to the spleen in a chicken model. Collectively, our data point to PAMP repression as a novel pathogenic mechanism to overcome the mucosal barrier through innate immune evasion.

## Author Summary

Some bacterial species contain pathogenic strains that are closely related genetically, but cause diseases that differ dramatically in their clinical presentation. One such species is *Salmonella enterica*, which contains non-typhoidal serotypes associated with a localized gastroenteritis and serotype Typhi (*S.* Typhi), the causative agent of a severe systemic infection termed typhoid fever. Conventional wisdom holds, that the ability of *S.* Typhi to overcome mucosal barriers and spread systemically in immunocompetent individuals evolved through acquisition of new virulence factors, which are absent from non-typhoidal *Salmonella* serotypes. Here, we demonstrate that acquisition of a regulatory gene, *tviA*, by *S*. Typhi alters expression of existing virulence factors (the flagellar regulon) such that molecular structures that are detected by the host innate immune are repressed after entering tissue. We propose that this mechanism contributes to innate immune evasion by *S*. Typhi, thereby promoting systemic dissemination.

## Introduction

Epithelial barriers form a first line of defense against microbial invasion. However, the ability to cross this physical barrier does not automatically result in systemic dissemination of the invading microbe. For example, non-typhoidal *Salmonella* serotypes, such as *Salmonella enterica* serotype Typhimurium (*S.* Typhimurium), invade the intestinal epithelium using the invasion associated type III secretion system (T3SS-1) [1] and employ a second type III secretion system (T3SS-2) to survive within tissue macrophages [2]. Despite the ability of non-typhoidal *Salmonella* serotypes to penetrate the epithelium and survive in macrophages, the infection remains localized to the terminal ileum, colon and mesenteric lymph node in immunocompetent individuals [3]. *S. enterica* serotype Typhi (*S.* Typhi) differs from non-typhoidal serotypes by its ability to cause a severe systemic infection in immunocompetent individuals termed typhoid fever [4]. However, little is known about the virulence mechanisms that enabled *S.* Typhi to overcome mucosal barrier functions and spread systemically, which is at least in part due to the lack of animal models for this strictly human adapted pathogen.

The chromosomes of *Salmonella* serotypes exhibit a high degree of synteny, which is interrupted by small insertions or deletions. One such insertion in *S.* Typhi is a 134 kb DNA region, termed *Salmonella* pathogenicity island (SPI) 7, which is absent from the *S.* Typhimurium genome and likely originates from a horizontal gene transfer event, as indicated by the presence of flanking tRNA genes [5]. Within SPI 7 lies a 14 kb DNA region, termed the *viaB* locus [6], which contains genes required for the regulation (*tviA*), the biosynthesis (*tviBCDE*), and the export (*vexABCDE*) of the Vi capsular antigen [7]. In addition to activating expression of the *S.* Typhi-specific Vi capsular antigen, the TviA protein represses important virulence factors that are highly conserved within the genus *Salmonella*. These include genes encoding flagella and T3SS-1, whose expression in *S.* Typhi is reduced by a TviA-mediated repression of the master regulator FlhDC [8]. However, the consequences of these changes in gene regulation for host pathogen interaction remain unclear.

Here we addressed the biological significance of TviA-mediated gene regulation. To explore how acquisition of a new regulatory protein impacted host microbe interaction, we determined whether introduction of the *tviA* gene into *S.* Typhimurium resulted in similar changes in gene expression as observed in *S.* Typhi. We then investigated how these TviA-mediated changes in gene expression affected the outcome of host interaction in an animal model, the chicken, in which *S.* Typhimurium causes a localized enteric infection.

## Results

### Changes in *S.* Typhimurium gene expression after chromosomal insertion of *tviA*

**Figure 1:**
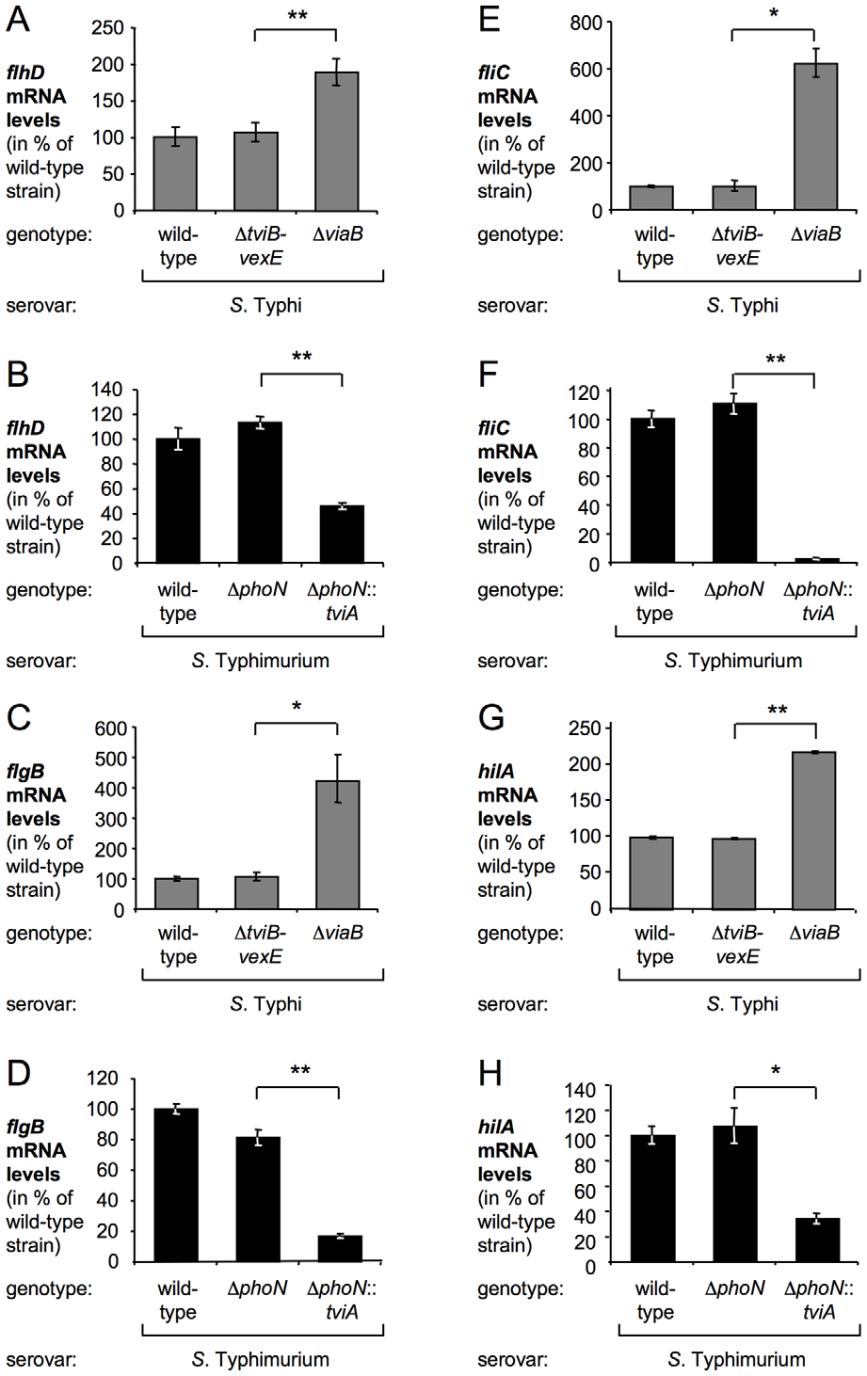
TviA represses flagellar and invasion gene expression in both *S*. Typhi and *S.* Typhimurium. *S*. Typhi (A, C, E, and G) and *S*. Typhimurium (B, D, F, and H) strains were grown under *tviA*-inducing conditions (SOB broth). Relative levels of *flhD* (A and B), *flgB* (C and D), *fliC* (E and F), and *hilA* (G and H) mRNA were measured by real-time qRT-PCR. The serotype and the genotype of the bacterial strains are indicated below each graph. Bars represent the geometric mean of three independent experiments ± standard error. Asterisks indicate the statistical significance of differences between data sets: * (*P*<0.05) or ** (*P*<0.01).

In *S*. Typhi, TviA-regulated genes have been identified and encompass the flagella regulon and genes encoding T3SS-1 [8]. To determine how TviA affects gene expression in a non-typhoidal serotype, the *tviA* gene was introduced into the *S.* Typhimurium chromosome and the gene expression profile compared to a published gene expression profile of TviA-regulated genes in *S.* Typhi [8]. Cluster analysis of gene expression profiles revealed that TviA influenced the transcription of similar regulatory circuits in *S.* Typhimurium and *S.* Typhi (Figure S1), including genes encoding regulatory, structural and effector components of the T3SS-1, and genes involved in chemotaxis, flagellar regulation and flagellar biosynthesis. To validate results obtained from gene expression profiling, relative transcription levels of genes encoding the flagellar regulator FlhD, the flagellar basal body protein FlgB, the flagellin FliC, and the T3SS-1 regulator HilA were determined in both serotypes by real-time qRT-PCR (Figure 1). Strains lacking the *tviA* gene (i.e. the *S.* Typhimurium wild-type strain, the *S.* Typhimurium Δ*phoN* mutant and the *S.* Typhi Δ*viaB* mutant) contained significantly higher mRNA levels of *hilA*, *flhD*, *flgB*, and *fliC* than observed in strains carrying the *tviA* gene (i.e. the *S.* Typhi wild-type strain, the *S.* Typhi Δ*tviB-vexE* mutant and the *S.* Typhimurium Δ*phoN*::*tviA* mutant, a strain in which the *phoN* gene had been replaced by the *tviA* gene).

### TviA alters expression of *S.* Typhimurium *flhC* and motility in response to osmolarity

**Figure 2:**
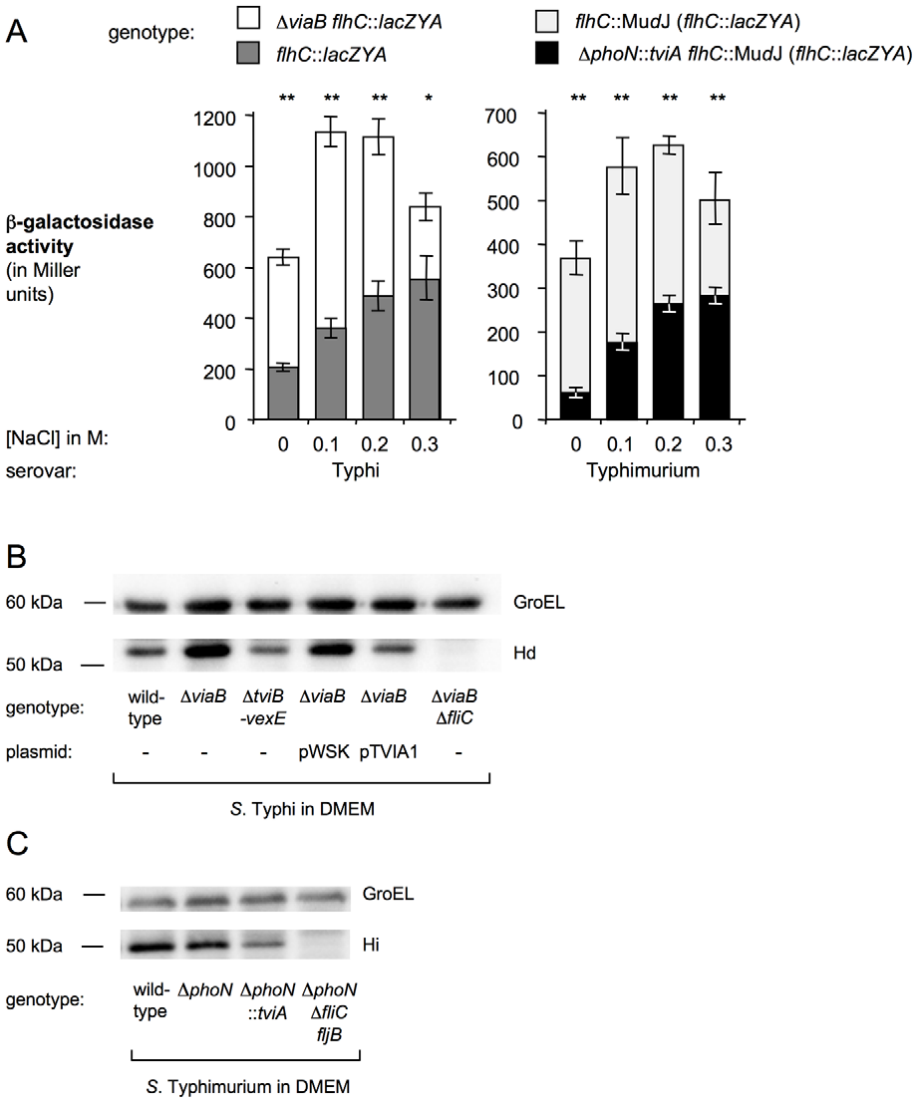
TviA represses flagellar gene expression under conditions mimicking tissue osmolarity. (A) A *S.* Typhi *flhC::lacZYA* mutant (SW197, dark gray bars), a *S.* Typhi Δ*viaB flhC*::*lacZYA* mutant (SW186, open bars), a *S.* Typhimurium *flhC::*Mu*d*J mutant (SW335, light gray bars) and a *S.* Typhimurium Δ*phoN*::*tviA flhC::*Mu*d*J mutant (SW316, black bars) were grown in tryptone yeast extract medium and β-galactosidase activity was measured. NaCl was added at the concentrations to increase osmolarity. Bars represent the geometric mean of three independent experiments ± standard error. Asterisks indicate statistical significance between data sets: * (*P*<0.05) or ** (*P*<0.01). (B) The *S*. Typhi wild-type strain (Ty2), a Δ*viaB* mutant (SW347), a Δ*tviB-vexE* mutant (SW74), and a Δ*viaB* Δ*fliC* mutant (SW483) were grown in tissue culture medium (DMEM) and FliC expression was detected by Western blot using H antiserum d. (C) The S. Typhimurium wild-type strain (IR715), a Δ*phoN* mutant (AJB715), a Δ*phoN*::*tviA* mutant (SW474) and a Δ*phoN* Δ*fliC fljB* mutant (SW681) were cultured in tissue culture medium (DMEM) and expression of FliC was detected by Western blot using *Salmonella* H antiserum i. Expression of GroEL was determined to ensure equal loading of samples, (αGroEL). Approximate position of standard proteins with known molecular mass is indicated.

Expression of the flagellum is controlled by the master regulator FlhDC (reviewed in [9]) and is reduced under low osmolarity in *S.* Typhi compared to *S.* Typhimurium [10]. Osmoregulation is mediated through the EnvZ/OmpR system in *S.* Typhi, which controls the availability of TviA. Under conditions of low osmolarity, TviA is expressed and represses *flhDC* transcription, thereby negatively regulating flagella biosynthesis [8], [11]. To understand the consequences of acquiring *tviA* by horizontal gene transfer, we determined whether differences in *flhDC* transcription between *S.* Typhi and *S.* Typhimurium could be fully accounted for by TviA-mediated gene regulation. Therefore, expression of *flhC* in *S.* Typhi and *S.* Typhimurium was monitored using transcriptional fusions to the *Escherichia coli lacZYA* reporter genes (Figure 2).

In the *S.* Typhi wild-type strain, *flhC* expression increased with increasing salt concentrations present in the culture medium (Figure 2A, dark gray bars). The *S.* Typhimurium wild-type strain exhibited a strikingly different *flhC* gene expression pattern, which peaked at medium salt concentrations (between 0.1 and 0.2 M NaCl) (Figure 2A, light gray bars). Removal of the *tviA* gene in the *S.* Typhi Δ*viaB* mutant resulted in an *flhC* gene expression pattern (Figure 2A, open bars) that was similar to that of the *S.* Typhimurium wild-type strain. Similarly, introduction of *tviA* into *S.* Typhimurium resulted in a *flhC* gene expression pattern (Figure 2A, closed bars) resembling that of the *S.* Typhi wild-type strain. TviA repressed motility under conditions of low osmolarity. Under conditions of high osmolarity (0.3 M NaCl), the presence or absence of the *tviA* gene did not alter motility in *S.* Typhi or *S.* Typhimurium, suggesting that TviA-mediated repression is relieved under this growth condition [8](Figure S2).

These observations suggested that the *tviA* gene is responsible for differences between *S.* Typhi and *S.* Typhimurium in expressing the master regulator of flagella expression and that the *tviA* gene product can be fully incorporated into the regulatory network existing in *S*. Typhimurium. Furthermore, these data supported the idea that TviA does not affect flagella expression under conditions of high osmolarity (Figure 2A), which are encountered in the intestinal lumen. In contrast, TviA repressed flagella expression under conditions that closely resembled the osmolarity encountered in human tissue.

### Repression of flagellin expression by TviA at tissue osmolarity

We next wanted to investigate whether TviA-mediated changes in gene transcription altered the amount of flagellin protein produced when *S.* Typhi strains were grown at an osmolarity encountered in tissue (i.e. after growth in DMEM tissue culture medium) (Figure 2B). Expression of the *S.* Typhi flagellin, FliC (also known as the *S.* Typhi Hd antigen), was monitored by Western blot (using anti Hd serum). Expression of the heat shock protein GroEL remained constant and was used as a loading control. In the presence of the *tviA* gene (i.e. in the *S.* Typhi wild-type strain or the *S.* Typhi Δ*tviB-vexE* mutant), a low level of FliC expression was detected when bacteria were grown under conditions mimicking tissue osmolarity (Figure 2B) or under conditions of low osmolarity (Figure S3). Deletion of *tviA* in *S.* Typhi (Δ*viaB* mutant) resulted in increased expression of FliC and introducing the cloned *tviA* gene (pTVIA1) restored FliC expression to wild-type levels.

Introduction of the *tviA* gene into the *S.* Typhimurium chromosome (Δ*phoN*::*tviA* mutant) reduced FliC (also known as the *S.* Typhimurium H1 or Hi antigen) protein levels when bacteria were grown in DMEM tissue culture medium (Figure 2C) or under conditions of low osmolarity (Figure S3). Expression of FljB, the H2 flagellin antigen of *S.* Typhimurium, was not detected by Western blot under conditions used in this study (data not shown). Collectively, these data suggested that TviA reduced the amount of FliC production in *S.* Typhi and *S.* Typhimurium under conditions of tissue osmolarity.

### TviA rapidly represses flagella expression in blood serum

**Figure 3:**
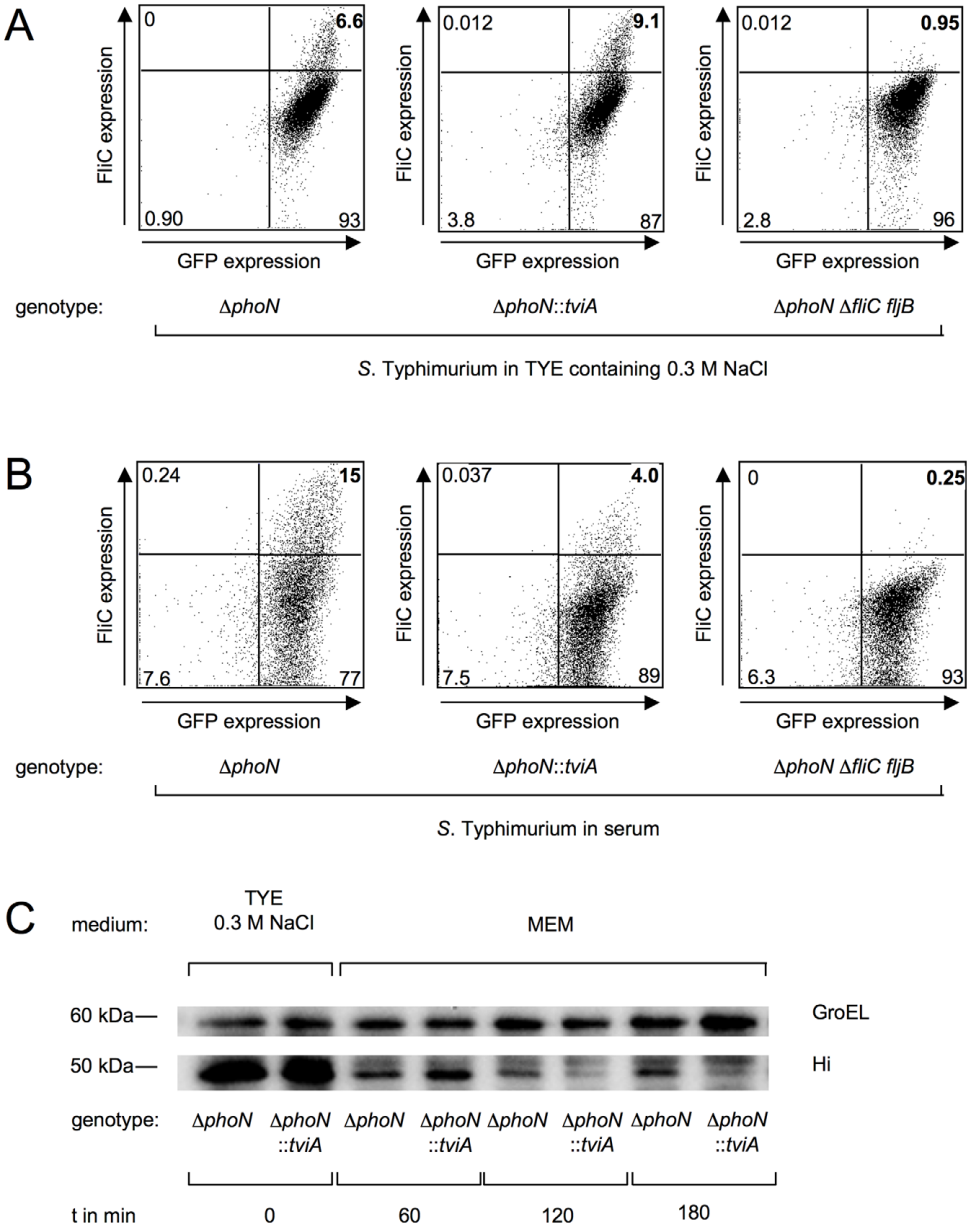
TviA is involved in a rapid decrease in flagella expression in serum. (A – B) A plasmid (pDW5) encoding GFP was introduced into *S*. Typhimurium strains. FliC expression of a *S*. Typhimurium Δ*phoN* mutant (AJB715[pDW5]), a Δ*phoN*::*tviA* mutant (SW474[pDW5]), and a Δ*phoN* Δ*fliC fljB* mutant (SW681[pDW5]) was detected by flow cytometry. Strains were grown for two hours in tryptone yeast extract broth (TYE) containing 0.3 M NaCl (A) or in murine serum (B). (C) Time dependent repression of FliC expression exerted by TviA. A S. Typhimurium Δ*phoN* mutant and a Δ*phoN*::*tviA* mutant were grown in tryptone yeast extract broth containing 0.3 M NaCl and subsequently transferred into tissue culture medium (MEM). FliC expression was detected by Western blot using *Salmonella* H antiserum i. Expression of GroEL was determined to ensure equal loading of samples (αGroEL). Approximate position of standard proteins with known molecular mass is indicated.

To further test this idea, we mimicked osmotic conditions encountered in the intestinal lumen or in tissue by suspending green fluorescent protein (GFP)-labeled bacteria in medium with high osmolarity or in serum, respectively. After a two-hour incubation, flagella expression was detected on the bacterial surface by flow cytometry. This analysis revealed that flagella were expressed by *S.* Typhimurium strains under osmotic conditions encountered in intestinal contents, regardless of the presence of *tviA* (Figure 3A). In contrast, TviA repressed flagellin expression under osmotic conditions encountered in serum, as indicated by a reduction of FliC on the surface of the strain carrying the *tviA* gene (i.e. the *S.* Typhimurium Δ*phoN*::*tviA* mutant) (Figure 3B).

Invasion of epithelial cells allows *Salmonella* to gain access to the lamina propria of the small intestine, a process that is accomplished in as little as two hours [12]. To test, whether *tviA* can repress flagellin expression within this time frame, the *S*. Typhimurium Δ*phoN* mutant and the Δ*phoN*::*tviA* mutant were grown under conditions of high osmolarity and subsequently shifted to osmolarity encountered in the tissue. Expression of FliC was determined at different time points by Western blot (Figure 3C). In comparison to the wild-type strain, the *tviA* gene product reduced the amount of flagellin expression as early as two hours after decreasing the osmolarity of the culture medium.

These data were consistent with the hypothesis that TviA does not alter gene expression in the intestinal lumen but rapidly (within two hours) represses flagellin expression upon bacterial entry into tissue.

### TviA-mediated flagellin regulation enables bacteria to evade sentinel functions of intestinal model epithelia

**Figure 4:**
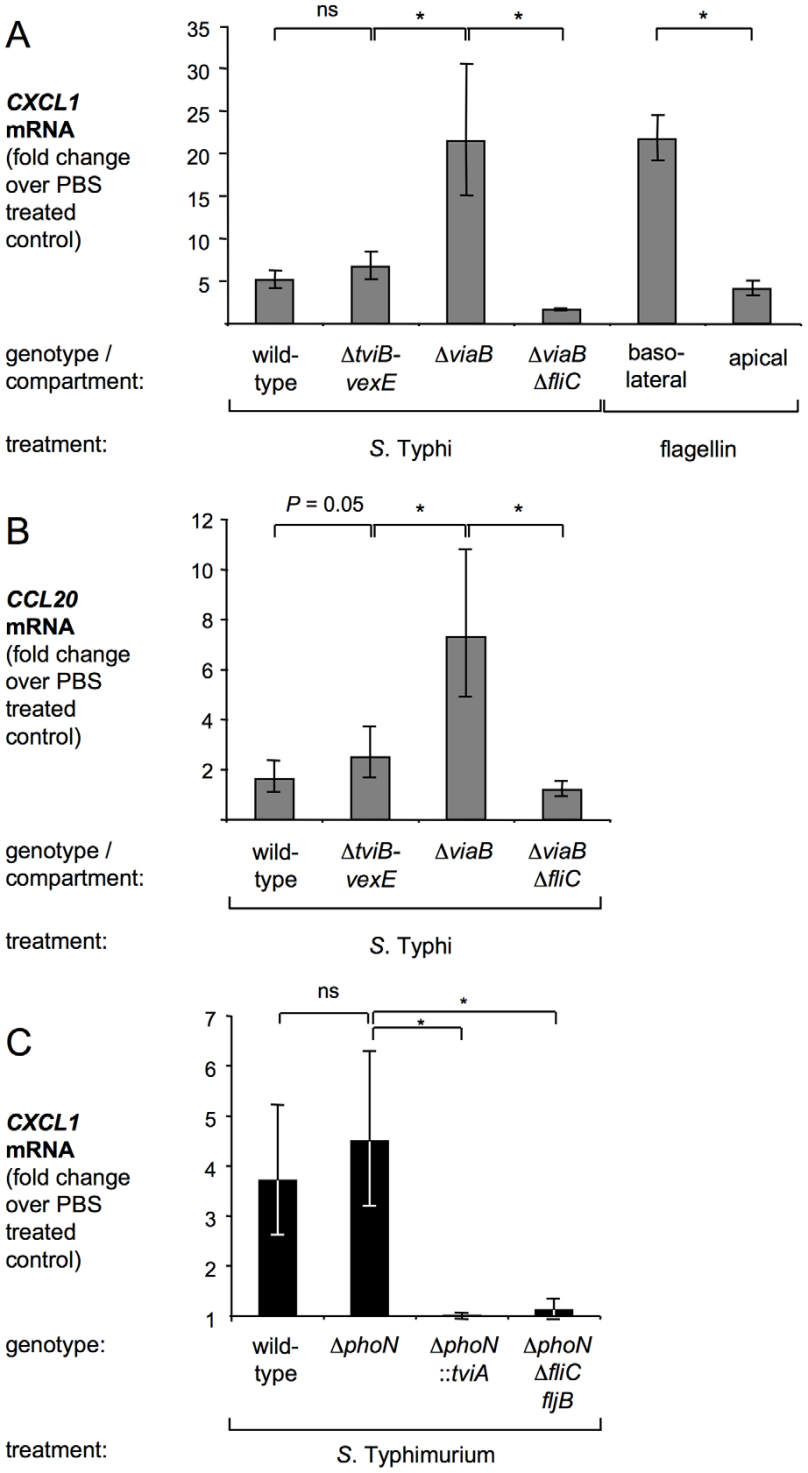
TviA-mediated flagellin repression in *S.* Typhi reduces the ability of model epithelia to serve as sentinels by detecting flagellin on their basolateral surface. (A and B) The *S*. Typhi wild-type strain (Ty2), a Δ*tviB-vexE* mutant (SW74), a Δ*viaB* mutant (SW347), and a Δ*viaB* Δ*fliC* mutant (SW483) were grown in tissue culture medium (MEM) and then added to the basolateral compartment of polarized T84 epithelial cells. Alternatively, purified flagellin was added to the basolateral or apical compartment as indicated. 3 hours later, relative expression of the chemokines *CXCL1* (A) and *CCL20* (B) was measured by real time qRT-PCR. (C) The *S*. Typhimurium wild-type strain (IR715), a Δ*phoN* mutant (AJ715), a Δ*phoN*::*tviA* mutant (SW474), and a Δ*phoN* Δ*fliC fljB* mutant (SW681) were grown in tissue culture medium (MEM) and then added to the basolateral compartment of polarized T84 epithelial cells. *CXCL1* transcript levels were quantified by real time qRT-PCR. Bars represent the geometric mean of three independent experiments ± standard error. Asterisks indicate the statistical significance of differences between data sets: * (*P*<0.05); ns: not statistically significant.

To mount responses that are appropriate to the threat, the innate immune system in the intestine needs to distinguish between harmless commensal bacteria that are present in the lumen and pathogenic microbes that invade tissue. One player in this process is the intestinal epithelium, which can discriminate between luminal commensals and invasive pathogens by a functional compartmentalization of Toll-like receptor (TLR) 5 expression. TLR5 is a pathogen recognition receptor specific for bacterial flagellin [13]. TLR5 is only expressed on the basolateral surface of the intestinal epithelium [14], [15]. Human colonic epithelial (T84) cells can be polarized to form a model epithelium that recapitulates the sentinel function of TLR5 in detecting bacterial translocation from the lumen [15], [16], [17]. We used this model to investigate whether TviA-mediated repression of flagellin expression in tissue is a mechanism to evade sentinel functions of model epithelia. The expression of *CCL20* (encoding the chemokine MIP-3α) and *CXCL1* (encoding the chemokine GROα) in polarized T84 cells was flagellin-dependent, as indicated by an absence of responses elicited by non-flagellated *S*. Typhi and *S*. Typhimurium mutants (Figure 4, S4, and Table S1). Furthermore, T84 model epithelia responded to basolateral, but not to apical stimulation with purified flagellin (Figure 4A), which was consistent with a functional compartmentalization of TLR5 expression reported previously [15]. Model epithelia were stimulated basolaterally with *S.* Typhi strains grown under conditions mimicking tissue osmolarity. The presence of *tviA* in the *S.* Typhi wild-type strain and the *S.* Typhi Δ*tviB-vexE* mutant resulted in a dramatic reduction in the relative transcript levels of *CXCL1* and *CCL20* (Figure 4A and B) compared to levels elicited by the *S.* Typhi Δ*viaB* mutant, which lacked the *tviA* gene.

To determine whether introduction of the *tviA* gene into *S.* Typhimurium would confer the ability to evade detection by model epithelia, polarized T84 cells were stimulated basolaterally with *S.* Typhimurium strains grown under conditions mimicking tissue osmolarity (Figure 4C). The absence of *tviA* in the *S.* Typhimurium wild-type strain and the *S.* Typhimurium Δ*phoN* mutant resulted in considerable higher mRNA levels of *CXCL1* in T84 cells compared to levels elicited by strains in which flagellin expression was repressed (*S.* Typhimurium Δ*phoN*::*tviA* mutant) or abrogated (*S.* Typhimurium Δ*phoN* Δ*fliC fljB* mutant). In summary, these data suggested that sentinel functions of the intestinal epithelium could be evaded by a TviA-mediated repression of flagellin expression in tissue.

### Expression of *tviA* in *S*. Typhimurium results in increased translocation to the spleen in a chicken model

By evading detection through sentinels of the intestinal immune system, TviA-mediated flagellin repression might prevent induction of mucosal barrier functions orchestrated by proinflammatory signals. Since our data pointed to a high degree of similarity between *S.* Typhi and *S.* Typhimurium in the mechanisms and consequences of TviA-mediated gene regulation, we reasoned that the relevance of TviA-mediated flagellin repression *in vivo* could be assessed using animal models of *S.* Typhimurium infection. The mouse model is not suited for this purpose, because *S.* Typhimurium rapidly disseminates to the liver and spleen of mice, suggesting that the pathogen can overcome mucosal barrier functions in this host species. In contrast, *S.* Typhimurium causes a localized gastroenteritis in immunocompetent individuals and is therefore susceptible to mucosal barrier functions encountered in humans. These barrier functions, which are present in humans but absent from mice, are specifically overcome by *S.* Typhi, as indicated by its ability of to cause typhoid fever. We thus reasoned that the consequences of TviA-mediated flagellin repression should be investigated in an animal, whose mucosal barrier functions, like the ones in humans, are sufficient for preventing systemic dissemination of *S.* Typhimurium. *S.* Typhimurium causes a localized enteric infection in chickens, an animal detecting flagellin expression through TLR5 [18], resulting in the activation of mucosal barrier functions [19]. This host was chosen for our analysis.

**Figure 5:**
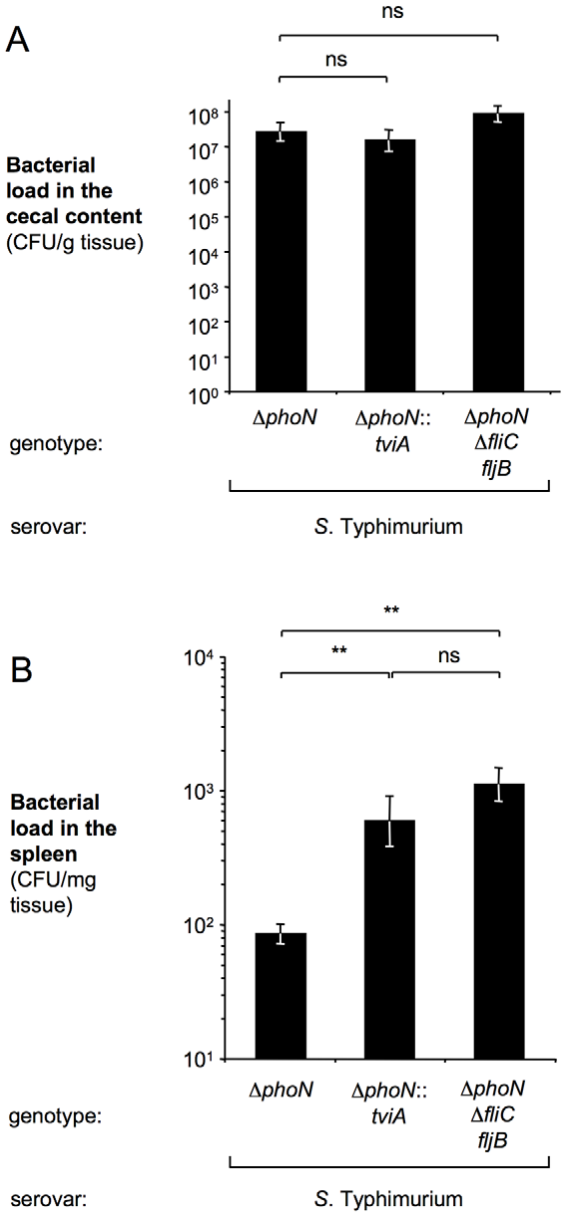
Introduction of the *tviA* regulatory gene into the *S*. Typhimurium chromosome promotes increased systemic dissemination in chickens. Groups of five 4-day-old chicks were inoculated orally with a *S*. Typhimurium Δ*phoN* mutant, a Δ*phoN*::*tviA* mutant or a Δ*phoN* Δ*fliC fljB* mutant. At 8 hours after inoculation, the bacterial load in the cecal contents (A) and the spleen (B) was determined. Bars represent the geometric mean ± standard error. Asterisks indicate the statistical significance of differences between data sets: ** (*P*<0.01); ns: not statistically significant.

Groups of four-day-old chickens were infected orally with the *S.* Typhimurium Δ*phoN* mutant, the *S.* Typhimurium Δ*phoN*::*tviA* mutant or the *S.* Typhimurium Δ*phoN* Δ*fliC fljB* mutant and bacterial translocation to the spleen was monitored at 8 hours after infection. The presence of the flagellin repressor TviA (Δ*phoN*::*tviA* mutant) or the absence of flagellin (Δ*phoN* Δ*fliC fljB* mutant) resulted in markedly increased systemic dissemination of *S.* Typhimurium compared to that observed with flagellated *S.* Typhimurium (Δ*phoN* mutant) (Figure 5). In contrast, no significant differences were detected between numbers of the *S.* Typhimurium Δ*phoN* mutant, the *S.* Typhimurium Δ*phoN*::*tviA* mutant or the *S.* Typhimurium Δ*phoN* Δ*fliC fljB* mutant recovered from intestinal contents. Since the flagellin proteins are among the most abundant proteins expressed by *S*. Typhimurium it was conceivable that TviA increased the growth rate by repressing the flagella regulon. However, the *tviA*-expressing strain (Δ*phoN*::*tviA* mutant) and the Δ*phoN* mutant were recovered in comparable numbers from the spleen of intraperitoneally infected mice 8 h after infection (Figure S5), indicating that TviA did not alter the growth rate of *S*. Typhimurium in tissue.

Taken together, these data were consistent with the idea that TviA-mediated repression of flagellin expression is a mechanism to overcome mucosal barrier functions, thereby promoting increased bacterial dissemination to the spleen.

## Discussion

The ability to cross epithelial linings is not sufficient for causing systemic bacterial dissemination in immunocompetent individuals, suggesting that additional barrier functions encountered in tissue successfully limit bacterial spread. At least some of these barrier functions are inducible by proinflammatory signals generated during bacterial translocation from the gut [20]. Here we provide support for the idea that evasion of inducible barrier functions by repressing a bacterial PAMP (i.e. flagellin) is a mechanism enhancing systemic bacterial dissemination from the intestine.

**Figure 6:**
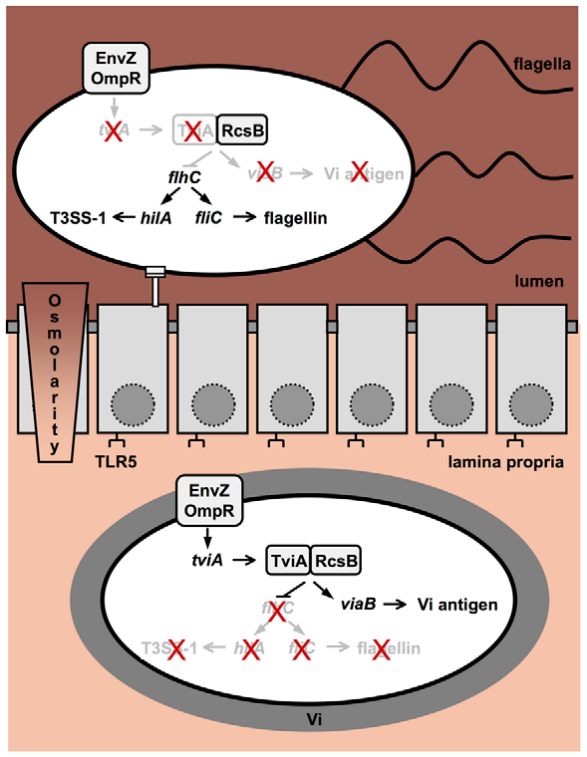
Proposed model of TviA-mediated changes in gene expression, which occur during the transition of bacteria from the intestinal lumen into tissue. Due to elevated osmolarity, *tviA* is not expressed in the intestinal lumen, allowing expression of invasion genes (T3SS-1) and flagella. In the lamina propria, a relative decrease in osmolarity leads to the expression of *tviA*. Under these conditions, TviA is available as a co-repressor for RcsB, resulting in reduced invasion gene and flagellar gene expression and activation of genes in the *viaB* locus, resulting in capsule (Vi antigen) production.

*S.* Typhimurium expresses flagellin during growth in the intestinal lumen as well as in Payers patch tissue, but flagellin expression ceases once bacteria disseminate to internal organs of mice, such as the spleen [21], [22]. Our data suggest that TviA-mediated flagellin repression is not operational in the intestinal lumen, but is rapidly initiated once bacteria encounter tissue osmolarity. The presence of TviA might therefore enable *S.* Typhi to more rapidly repress flagellin expression upon invasion of the intestinal mucosa (Figure 6) compared to *S.* Typhimurium, which still expresses flagellin in intestinal tissue [21]. Bacterial translocation across the epithelial barrier into the underlying tissue is observed within 2 hours after infection of ligated ileal loops with *S.* Typhimurium [12], [23]. TviA markedly reduced flagellin repression within 2 hours of bacterial growth at an osmolarity encountered in tissue. TviA-mediated flagellin repression thus occurred within the time frame required for bacterial translocation across an epithelial barrier *in vivo*. Similarly, TviA activates expression of the Vi capsular antigen when *S.* Typhi transits from the intestinal lumen into tissue in a ligated ileal loop model [24].

Expression of flagellin by bacteria arriving in tissue is of consequence, because sentinels monitoring microbial translocation from the gut can detect this PAMP. One of the mechanisms by which the intestinal mucosa distinguishes luminal bacteria from bacteria in tissue can be recapitulated using polarized T84 intestinal epithelial cells, which express TLR5 only on their basolateral surface [15], [17]. Here we show that TviA-mediated flagellin repression enabled bacteria to evade this sentinel function of epithelial cells. It is possible that other cell types may contribute to detecting flagella *in vivo*. However, regardless of the mechanism(s) by which flagellin stimulates innate immunity in the intestine, our results demonstrate that TviA-mediated flagellin repression resulted in increased bacterial dissemination to the spleen of chickens. The idea that detection of flagella contributes to barrier function is also consistent with the finding that a non-flagellated *S.* Typhimurium *fliM* mutant exhibits an enhanced ability to establish systemic infection in chickens compared to the wild-type strain [19]. It may therefore not be a coincidence that *S. enterica* serotype Gallinarum (*S.* Gallinarum), the only serotype associated with a severe systemic infection in chickens [25], does not express flagella. Similarly, tight regulation of flagellin expression is required for virulence of *Yersinia enterocolitica* in mice [26].

It should be pointed out, however, that evading detection of flagella by the innate immune system, although necessary, might not be sufficient for causing systemic disease. For example, *Shigella* species cause a localized colitis in humans, despite the fact that these pathogens do not express flagellin. A possible explanation for the lower propensity of *Shigella* species to cause systemic infection is the absence of a *Salmonella* T3SS-2 equivalent. T3SS-2 is a *Salmonella* virulence factor important for macrophage survival [2], [27], and its absence in *Shigella* species may render these pathogens more vulnerable to phagocyte attack. In turn, T3SS-2 may be necessary, but it is not sufficient for systemic dissemination, because *S.* Typhimurium, which carries this virulence factor, causes a localized infection in immunocompetent individuals. Thus, the ability of *S.* Typhi to cause systemic disease in humans likely evolved by combining virulence factors conserved among *Salmonella* serotypes (e.g. T3SS-2 and others) with newly acquired virulence traits (e.g. TviA-mediated flagellin repression and others).

The picture emerging from these studies is that the presence in *S.* Typhi of a regulator, TviA, which senses the transition of bacteria from the intestinal lumen into tissue, enables the pathogen to rapidly cease flagellin expression when crossing the epithelial lining, thereby preventing the induction of barrier functions that limit bacterial dissemination (Figure 6). At the same time, TviA induces expression of the Vi capsular antigen [24], a virulence factor preventing detection of the pathogen through TLR4 [28]. Collectively, these mechanisms interfere with innate immune surveillance at the mucosal surface [17], [29], [30], [31], resulting in reduced intestinal inflammation [32], [33] and contributing to increased dissemination. It should be pointed out that overcoming barrier functions through TviA-mediated regulation is not sufficient for causing typhoid fever, because subsequent to its initial systemic spread, *S.* Typhi requires additional virulence mechanisms to establish residence in internal organs, persist and, after a two-week incubation period, cause disease.

## Materials and Methods

### Bacterial strains, plasmids and culture conditions

Bacterial strains and plasmids used in this study are listed in table 1. *Salmonella* strains were routinely grown aerobically at 37°C in Luria Bertani (LB) broth (10 g/l tryptone, 5 g/l yeast extract, 10 g/l NaCl) or on LB agar plates. To induce optimal expression of TviA, strains were grown overnight in LB, diluted in either Super Optimal Broth (SOB) (20 g/liter tryptone, 5 g/liter yeast extract, 10 mM NaCl, 2.5 mM KCl, 10 mM MgCl_2_) [29] or tryptone yeast extract broth (10 g/l tryptone, 5 g/l yeast extract) and aerobically grown to mid-log phase at 37°C. When appropriate, antibiotics were added at the following concentrations: chloramphenicol 0.03 mg/ml, carbenicillin 0.1 mg/ml, and kanamycin 0.05 mg/ml.

**Table 1:** Bacterial strains and plasmids used in this study.

*S*. Typhimurium		
IR715	nalidixic acid-resistant derivative of S. Typhimurium strain ATCC 14028	[43]
AJB715	IR715 Δ*phoN*::Kan^r^	[44]
SPN313	IR715 Δ*fliC*(−25 to +1494) *fljB5001*::Mu*d*Cm	[45]
SW124	IR715 (pWSK29)	[11]
SW125	IR715 (pTVIA1)	[29]
SW316	IR715 Δ*phoN*::*tviA* Cm^r^ *flhC5456*::Mu*d*J	[8]
SW335	IR715 *flhC5456*::Mu*d*J	This study
SW474	IR715 Δ*phoN*::*tviA* Cm^r^	[8]
SW681	IR715 Δ*phoN*::Kan^r^ Δ*fliC*(−25 to +1494) *fljB5001*::Mu*d*Cm	This study
TH4054	LT2 *flhC5456*::Mu*d*J	[46]
*S*. Typhi		
Ty2	wild-type strain, Vi^+^	ATCC 700931
STY2	Ty2 Δ*viaB*::Kan^r^, Vi^−^	[29]
SW74	Ty2 Δ*tviB-vexE*::Cm^r^, Vi^-^	[11]
SW186	Ty2 Δ*viaB*::Kan^r^ *flhC*::pSW63 (*flhC*::*lacZYA*, Cm^r^)	[11]
SW197	Ty2 *flhC*::pSW63 (*flhC*::*lacZYA*, Cm^r^)	[11]
SW347	Ty2 Δ*viaB*, Vi^−^	[8]
SW359	Ty2 Δ*fliC*(−25 to +1494)	[8]
SW483	Ty2 Δ*viaB* Δ*fliC*(−25 to +1494)	[8]
Plasmids		
pWSK29	*ori*(pSC101) *bla*	[47]
pTVIA1	*tviA* under control of its native promoter in pWSK29, *ori*(pSC101) *bla*	[29]
pDW5	P*_tetA_*-*gfp* in pBR322	[21]

*Cm^r^: Chloramphenicol resistance; Kan^r^: Kanamycin resistance.

### P22 mediated generalized transduction

Phage P22 HT int-105 was used for transduction as described previously[34], [35]. To construct strain SW335, a P22 lysate of strain TH4054 was used to transduce the *flhC5456*::Mu*d*J mutation into IR715. SW681 was constructed by transducing the Δ*phoN*::Kan^r^ mutation of the strain AJB715 into SPN313.

### Purification of bacterial RNA

Bacterial RNA was isolated as described previously [8]. Briefly, *Salmonella* strains were statically grown in 5 ml SOB broth for 2 h. 0.8 ml of a 5% phenol solution (in ethanol) was added and the bacterial cells collected by centrifugation. The pellet was resuspended in 0.4 ml 0.1 mg/ml lysozyme, 1 mM ethylenediaminetetraacetic acid (EDTA) 10 mM, Tris/Cl pH 8.0 and incubated at room temperature for 30 min. Cells were lysed by adding 40 µl 10% sodium dodecyl sulfate (SDS). 0.44 ml 1 M sodium acetate as well as 0.9 ml hot (65°C) phenol was added to the sample and the emulsion was incubated at 65°C for 6 min, incubated on ice for 10 min and centrifuged at 20,000 g for 10 min at 4°C. The upper phase was extracted with 0.9 ml chloroform. After centrifugation at 20,000 g for 5 min at 4°C, the RNA was precipitated by adding 80 µl 1 mM EDTA 3 M sodium acetate pH 5.2 and 1 ml isopropanol. Samples were centrifuged for 30 min at 20,000 g at 4°C and the RNA pellet was washed with 1 ml 80% Ethanol. The air-dried RNA was resuspended in RNase-free water and traces of genomic DNA were removed by rigorous DNase treatment according to the recommendation of the manufacturer (DNA-free DNase treatment, Applied Biosystems).

### Gene expression profiling

Gene expression profiling experiments of the *S*. Typhimurium strains SW124 and SW125 were conducted identically to experiments described previously [8]. Briefly, RNA was extracted from one bacterial culture grown statically in 5 ml SOB broth until the turbidity reached an optical density of OD_600_ = 0.4−0.5. Microarray hybridization and scanning steps were performed by the UC Davis ArrayCore Microarry facility as described previously [36] with the modifications described in [8]. The TM4 Microarray Software Suite [37] was used for data processing and analysis as described previously [8]. Data from the reference data set (*S*. Typhi, [8]) was averaged and a cluster analysis of the gene expression profile of *S*. Typhi and *S*. Typhimurium was performed by the Clustering Affinity Search Technique (CAST) algorithm [38], [39] (initial threshold parameter of 0.85). Genes identified to be regulated by TviA in *S*. Typhi and *S*. Typhimurium are listed in supplementary table S1. Microarray data have been deposited at the Gene Expression Omnibus database under the accession number GSE20321.

### SDS-PAGE and Western blot

Expression of flagellin was determined by Western blot as described previously [8]. In brief, *Salmonella* strains were grown aerobically for 2 h at 37°C in Dulbecco's Modified Eagle Medium (DMEM) (Invitrogen). For time course experiments, *Salmonella* strains were grown for 16 h in tryptone yeast extract broth containing 0.3 M NaCl and diluted in Minimum Essential Medium Eagle (MEM) medium (Invitrogen). Culture turbidity (OD_600_) was measured and bacterial cells were lysed in loading buffer (50 mM Tris/HCl, 100 mM dithiothreitol, 2% SDS, 0.1% bromophenol blue, 10% glyerol). A portion of the lysate corresponding to approximately 5×10^7^ colony forming units (CFU) was resolved by SDS-polyacrylamide gel electrophoresis (PAGE) [40]. Proteins were transferred onto a polyvinylidene fluoride membrane (Millipore) using a semi-dry transfer system (Bio-Rad laboratories). To detect FliC and GroEL expression, rabbit *Salmonella* H antiserum d (Difco), *Salmonella* H antiserum i (Difco), and anti-GroEL antiserum (Sigma), respectively, as well as a horse radish peroxidase-conjugated goat anti-rabbit secondary antibody (Bio-Rad laboratories) were used. Chemiluminescence (SuperSignal West Pico Chemiluminescent Substrate, Thermo Scientific) was detected by a BioSpectrum Imaging System (UVP) and images were processed in Photoshop CS2 (Adobe) to adjust brightness levels.

### β-Galactosidase assay

*Salmonella* strains were grown overnight in tryptone yeast extract broth, diluted 1∶50 in 5 ml tryptone yeast extract broth and incubated for 3 h at 37°C. To adjust the osmolarity, NaCl was added to the media of the subculture as indicated. β-Galactosidase activity was measured as described previously [8], [41]. The experiment was performed in triplicate.

### Detection of flagella expression by flow cytometry

Strains were grown overnight in LB broth, diluted 1∶50 in fresh LB and incubated at 37°C until log phase. 5×10^8^ CFU were re-suspended in either 0.05 ml of mouse serum or in 0.05 ml of tryptone yeast extract broth containing 0.3 M NaCl and incubated for 2 hours at 37°C. Bacteria were cellected by centrifugation at 6000 g for 5 min at room temperature. Pellets were washed twice in fluorescence activated cell sorting (FACS) buffer (1% Bovine serum albumin in phosphate buffered saline [PBS]) and re-suspended in 0.1 ml of FACS buffer. Polyclonal rabbit anti-FliC was added and incubated on ice for 30 minutes. A secondary R-PE conjugated goat-anti rabbit (Jackson ImmunoResearch) was added and incubated on ice for 30 minutes. Bacteria were fixed in 4% Formalin for 1 hour and analyzed using an LSR II flow cytometer (Beckton-Dickinson). Results were analyzed using FlowJo software (Treestar).

### Stimulation of polarized T84 cells

The colonic carcinoma cell line T84 was obtained from the American Type Culture Collection (ATCC, CCL-248). T84 cells were routinely maintained in DMEM-F12 medium containing 1.2 g/l sodium bicarbonate, 2.5 mM L-glutamine, 15 mM 4-(2-hydroxyethyl)-1-piperazineethanesulfonic acid (HEPES), 0.5 mM sodium pyruvate (Invitrogen), and 10% fetal bovine serum (FBS; Invitrogen). To polarize T84 cells, cells were seeded at a density of 1×10^6^ cells per well in the apical compartment of transwell plates (12 mm diameter, pore size 0.4 µm) (Corning) and incubated for 5 to 10 days until the transepithelial electrical resistance exceeded a value of 1.5 kΩcm^2^. Media in both compartments was replaced every second day.

*Salmonella* strains were grown over night at 37°C in LB, diluted 1∶50 in yeast extract broth or MEM medium (Invitrogen) and incubated for 2 h 30 min at 37°C with aeration. T84 cells were activated by adding 2×10^6^ CFU into the basolateral compartment containing 1 ml of media. Purified *Salmonella* flagellin (InvivoGen) was added into the indicated compartment at a concentration of 1 µg/ml. After 3 h, eukaryotic RNA was isolated as described previously [11] using TRI reagent (Molecular Research Center) In brief, cells were lysed in 0.5 ml TRI reagent and this homogenate extracted with 0.1 ml chloroform (Sigma). The suspension was centrifuged at 12,000 g for 15 min. Nucleic acids were precipitated from the aqueous phase by adding 0.25 ml isopropanol (Sigma) and by centrifugation at 12,000 g for 8 min. The RNA pellet was washed with 75% Ethanol, air-dried and resuspended in water. Traces of DNA were removed by DNase treatment according to the recommendation of the manufacturer (DNA-free DNase treatment, Applied Biosystems).

### Real-time qRT-PCR

Real-time quantitative (q) reverse transcriptase (RT)-polymerase chain reaction (PCR) was performed as described previously [11]. 1 µg of DNase treated bacterial or eukaryotic RNA served as a template for RT-PCR in a 50 µl volume. Random hexamer dependent amplification was performed according to the recommendations of the manufacturer (TaqMan reverse transcription reagents; Applied Biosystems). SYBR Green (Applied Biosystems) based real-time PCR was performed in an 11 µl volume employing 4 µl of cDNA as a template. Primers are listed in table 2 and were added at a final concentration of 250 nM. Primers used to detect expression of bacterial genes were designed to amplify targets from both *Salmonella* serotypes with equal efficiency. Data was acquired by a GeneAmp 7900 HT Sequence Detection System (Applied Biosystems) and analyzed using the comparative Ct method (Applied Biosystems). Bacterial gene transcription in each sample was normalized to the respective levels of guanylate kinase mRNA, encoded by the *gmk* gene. Eukaryotic gene expression was normalized to the respective levels of *GAPDH* mRNA.

**Table 2:** Primers used for real time PCR in this study.

Organism	Target gene	Sequence	Reference
*S. enterica*	*gmk*	5′-TTGGCAGGGAGGCGTTT-3′ 5′-GCGCGAAGTGCCGTAGTAAT-3′	[48]
	*flhD*	5′-ACAGCGTTTGATCGTCCAG-3′ 5′-GTTTGCCATCTCTTCGTTGA-3′	This study
	*flgB*	5′-GCAGTTTGCGGATAACAGTC-3′ 5′-TCCTCCCTGTAGCACATTCA-3′	This study
	*fliC*	5′-GTAACGCTAACGACGGTATC-3′ 5′-ATTTCAGCCTGGATGGAGTC-3′	[8]
	*hilA*	5′-ATTAAGGCGACAGAGCTGGA-3′ 5′-GAATAGCAAACTCCCGACGA-3′	[8]
*H. sapiens*	*GAPDH*	5′-CCAGGAAATGAGCTTGACAAAGT-3′ 5′-CCCACTCCTCCACCTTTGAC-3′	[29]
	*CCL20* (*MIP3A*)	5′-CTGCTTTGATGTCAGTGCTGCTAC-3′ 5′-CTGCCGTGTGAAGCCCACAATAAA-3′	[49]
	*CXCL1 (GROA)*	5′-TGCGCCCAAACCGAAG-3′ 5′-TGCAGGATTGAGGCAAGCTT-3′	[50]

### Experimental infections of chickens

All procedures described in this study were conducted as described previously [42]. Briefly, specific pathogen free eggs were obtained from Charles River (North Franklin, CT). Eggs were kept in an egg incubator at 38°C and a humidity of 58–65% for 21 days and were periodically rolled for the first 18 days. Chickens were housed in a poultry brooder (Alternative Design Manufacturing, Siloam Springs, AR) at a temperature of 32°C to 35°C. Tap water and irradiated lab chick diet (Harlan Teklad, Madison, WI) was provided *ad libitum*. *S*. Typhimurium strains were grown aerobically at 42°C for 16 h in LB broth. Fifteen 4-day-old, unsexed White Leghorn chicks were orally inoculated in groups of five with either 1×10^9^ CFU of the *S*. Typhimurium strains AJB715, SW474, or SW681 in 0.1 ml LB broth. Animals were euthanized by asphyxiation with CO_2_ 8 h after inoculation. The spleen and a sample of the cecal content were homogenized in sterile PBS and serial ten-fold dilutions spread on LB agar plates containing the appropriate antibiotics.

### Experimental infections of mice

C57BL/6 mice were obtained from The Jackson Laboratory. Animals were housed under specific-pathogen-free conditions and provided with water and food *ad libitum*. *S*. Typhimurium strains were grown aerobically for 16 h at 37°C. Groups of 4 female mice (10 to 11 weeks of age) were injected intraperitoneally with 1×10^6^ CFU of the *S*. Typhimurium strains IR715, AJB715, or SW474 suspended in PBS. 8 h after infection, animals were euthanized and the spleen collected. Serial 10-fold dilutions of the splenic homogenate were spread on LB agar plates containing nalidixic acid.

### Statistical analysis

For the statistical analysis of ratios (i.e. increases in gene expression), values were transformed logarithmically for further statistical analysis. Data presented in bar graphs are geometric means +/− standard error. A parametric test (Student's *t*-test) was used to determine whether differences between treatment groups were statistically significant (*P*<0.05). For data from tissue culture experiments and gene expression analysis, paired statistical analysis was used.

### Ethics statement

All animal experiments were performed according to Association for Assessment and Accreditation of Laboratory Animal Care (AAALAC) guidelines. Experimental procedures with chickens were approved by the Texas A&M University Institutional Animal Care and Use Committee (IACUC). All experimental procedures with mice were approved by the UC Davis IACUC.

## Supporting Information

Figure S1 TviA regulates similar gene clusters in *S*. Typhimurium (Tm) and *S.* Typhi (Ty). Gene expression profiling was performed on bacterial strains (*S.* Typhimurium IR715 (pWSK29), *S.* Typhimurium IR715 (pTVIA1), *S.* Typhi Δ*viaB* mutant, and *S.* Typhi Δ*tviB-vexE* mutant) grown under low osmolarity condition (SOB broth). Similarities in gene expression between samples were identified using a CAST algorithm. Bars above the heat maps represent geometric means of fold change ± standard deviation for cluster of genes repressed (clusters 1 - 5) or activated (clusters 6 and 7) by TviA. The number of genes with no change in gene expression (cluster 8) is indicated. The number of genes within each cluster is indicated below each heat map. A black colored heat map indicates either no change in gene expression or no gene expression detected.
(0.97 MB TIF)

Figure S2 TviA regulates motility in response to osmolarity. Motility plates containing 10 g/l tryptone and 0.3% agar were inoculated with the indicated bacterial strains and incubated at 37°C for 24 h (*S*. Typhi) or 7 h (*S*. Typhimurium). In experiments shown in the upper panel, NaCl was added at a concentration of 300 mM to increase the osmolarity of the medium as indicated on the left. Experiments were performed in triplicate, of which only one representative image is shown.
(0.45 MB TIF)

Figure S3 Flagellin represses FliC expression in medium with low osmolarity. The indicated S. Typhi and S. Typhimurium strains were cultured aerobically for 2 h at 37°C in tryptone yeast extract broth containing 0.05 M NaCl. Expression of FliC was detected by Western blot using *Salmonella* H antiserum d (*S*. Typhi) or i (*S*. Typhimurium). Expression of GroEL was determined to ensure equal loading of samples, (αGroEL). Approximate position of standard proteins with known molecular mass is indicated.
(0.36 MB TIF)

Figure S4 TviA-mediated flagellin repression in *S.* Typhi reduces chemokine expression in polarized T84 cells. The *S*. Typhi wild-type strain (Ty2), a Δ*tviB-vexE* mutant (SW74), a Δ*viaB* mutant (SW347), and a Δ*fliC* mutant (SW359) were grown in tryptone yeast extract broth for 150 min and added to the basolateral compartment of polarized T84 epithelial cells. Alternatively, purified flagellin was added to the basolateral or apical compartment as indicated. 3 h later, relative expression of the chemokines *CXCL1* (top panel) and *CCL20* (bottom panel) was measured by real time qRT-PCR. Bars represent the geometric mean of three independent experiments ± standard error. Asterisks indicate the statistical significance of differences between data sets: * (*P* < 0.05) or ** (*P* < 0.01); ns: not statistically significant.
(0.31 MB TIF)

Figure S5 Effect of TviA on growth rate in tissue. Groups of four mice were infected intraperitoneally with the *S*. Typhimurium wild-type strain (IR715), the Δ*phoN* mutant (AJB715), and the Δ*phoN*::*tviA* mutant (SW474) and the bacterial load in the spleen determined eight hours after infection. Bars represent the geometric mean of three independent experiments ± standard error.
(0.17 MB TIF)

Table S1 Comparison of TviA-regulated genes in *S.* Typhi and *S.* Typhimurium.
(0.10 MB PDF)
