# Supplementary figures and images for: A Rapid Change in Virulence Gene Expression during the Transition from the Intestinal Lumen into Tissue Promotes Systemic Dissemination of Salmonella
Source: PLoS Pathog. 2010 Aug 19;6(8):e1001060. doi: 10.1371/journal.ppat.1001060 (PMC2924370; doi:10.1371/journal.ppat.1001060)

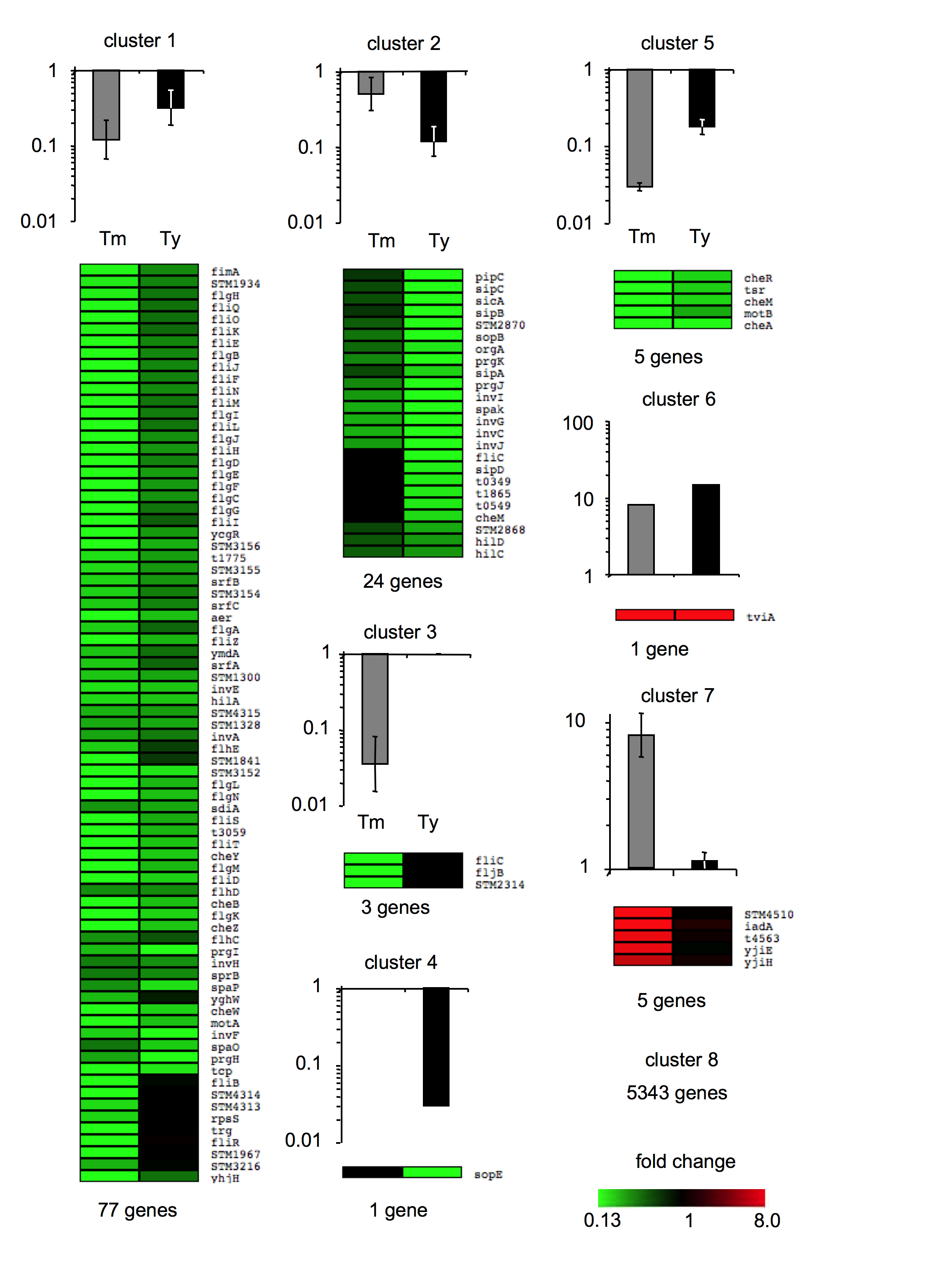

Supplement: Figure S1 — TviA regulates similar gene clusters in S. Typhimurium (Tm) and S. Typhi (Ty). Gene expression profiling was performed on bacterial strains (S. Typhimurium IR715 (pWSK29), S. Typhimurium IR715 (pTVIA1), S. Typhi ΔviaB mutant, and S. Typhi ΔtviB-vexE mutant) grown under low osmolarity condition (SOB broth). Similarities in gene expression between samples were identified using a CAST algorithm. Bars above the heat maps represent geometric means of fold change ± standard deviation for cluster of genes repressed (clusters 1 - 5) or activated (clusters 6 and 7) by TviA. The number of genes with no change in gene expression (cluster 8) is indicated. The number of genes within each cluster is indicated below each heat map. A black colored heat map indicates either no change in gene expression or no gene expression detected. (0.97 MB TIF) [file ppat.1001060.s001.tif]

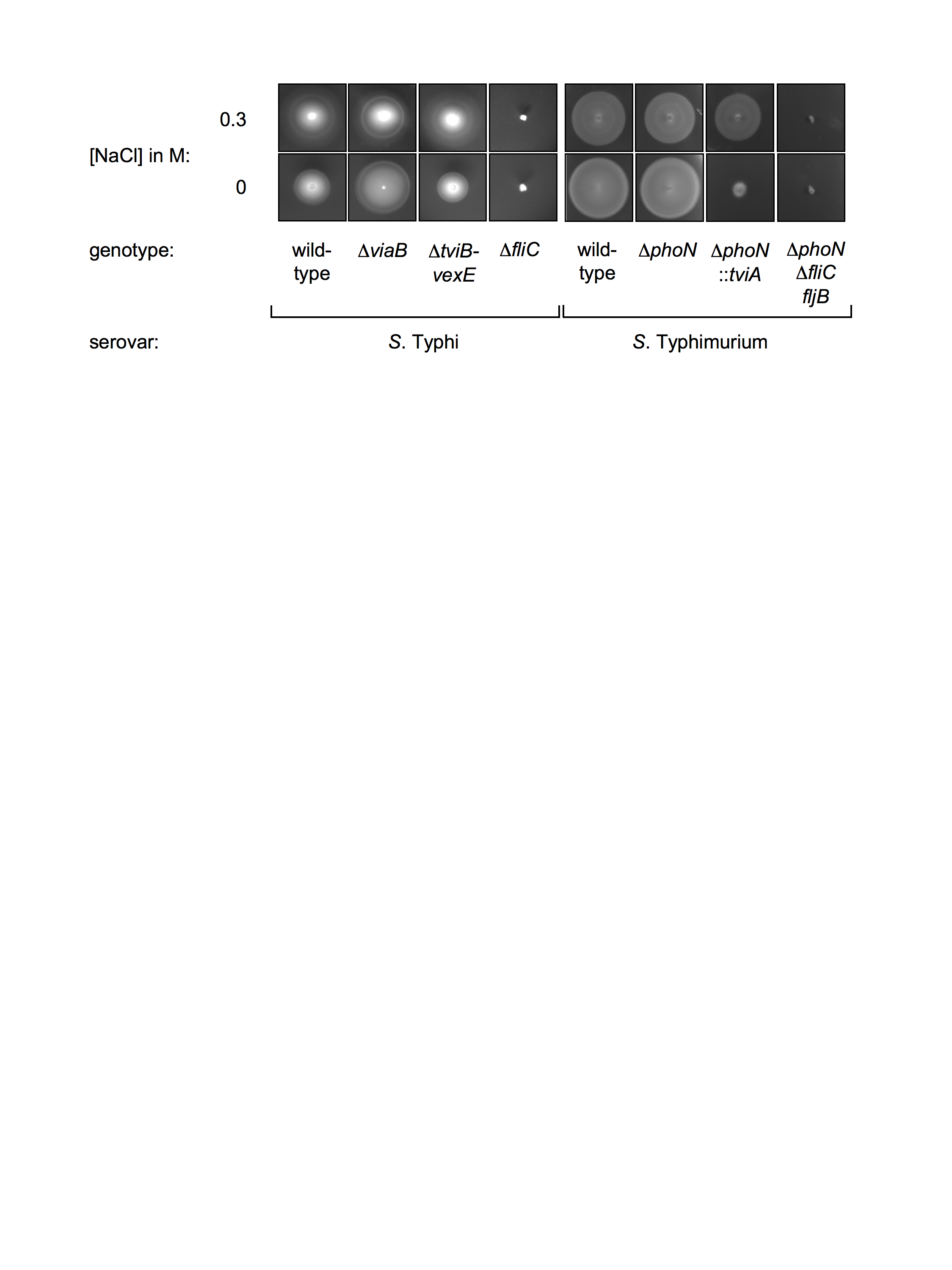

Supplement: Figure S2 — TviA regulates motility in response to osmolarity. Motility plates containing 10 g/l tryptone and 0.3% agar were inoculated with the indicated bacterial strains and incubated at 37°C for 24 h (S. Typhi) or 7 h (S. Typhimurium). In experiments shown in the upper panel, NaCl was added at a concentration of 300 mM to increase the osmolarity of the medium as indicated on the left. Experiments were performed in triplicate, of which only one representative image is shown. (0.45 MB TIF) [file ppat.1001060.s002.tif]

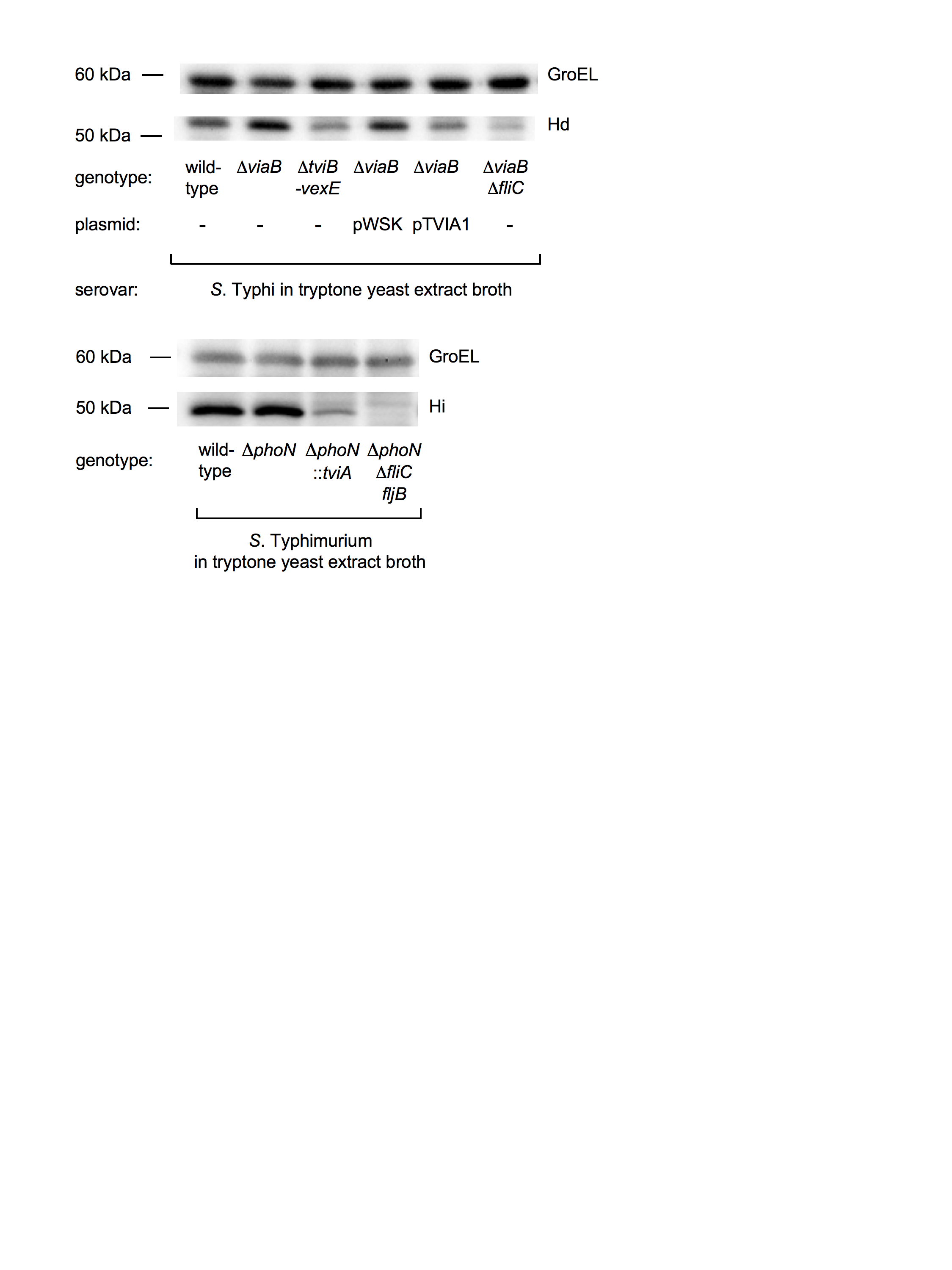

Supplement: Figure S3 — Flagellin represses FliC expression in medium with low osmolarity. The indicated S. Typhi and S. Typhimurium strains were cultured aerobically for 2 h at 37°C in tryptone yeast extract broth containing 0.05 M NaCl. Expression of FliC was detected by Western blot using Salmonella H antiserum d (S. Typhi) or i (S. Typhimurium). Expression of GroEL was determined to ensure equal loading of samples, (αGroEL). Approximate position of standard proteins with known molecular mass is indicated. (0.36 MB TIF) [file ppat.1001060.s003.tif]

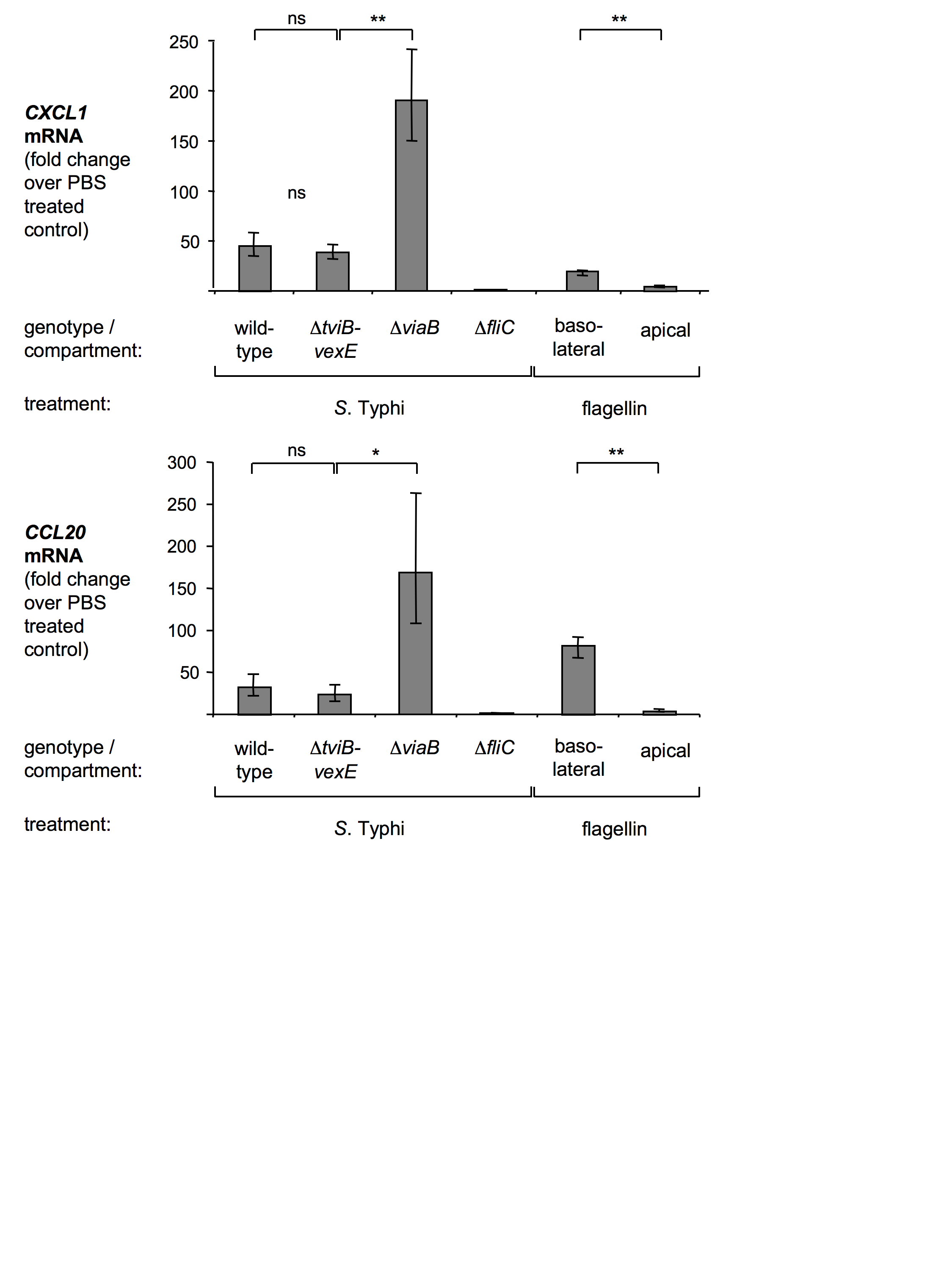

Supplement: Figure S4 — TviA-mediated flagellin repression in S. Typhi reduces chemokine expression in polarized T84 cells. The S. Typhi wild-type strain (Ty2), a ΔtviB-vexE mutant (SW74), a ΔviaB mutant (SW347), and a ΔfliC mutant (SW359) were grown in tryptone yeast extract broth for 150 min and added to the basolateral compartment of polarized T84 epithelial cells. Alternatively, purified flagellin was added to the basolateral or apical compartment as indicated. 3 h later, relative expression of the chemokines CXCL1 (top panel) and CCL20 (bottom panel) was measured by real time qRT-PCR. Bars represent the geometric mean of three independent experiments ± standard error. Asterisks indicate the statistical significance of differences between data sets: * (P < 0.05) or ** (P < 0.01); ns: not statistically significant. (0.31 MB TIF) [file ppat.1001060.s004.tif]

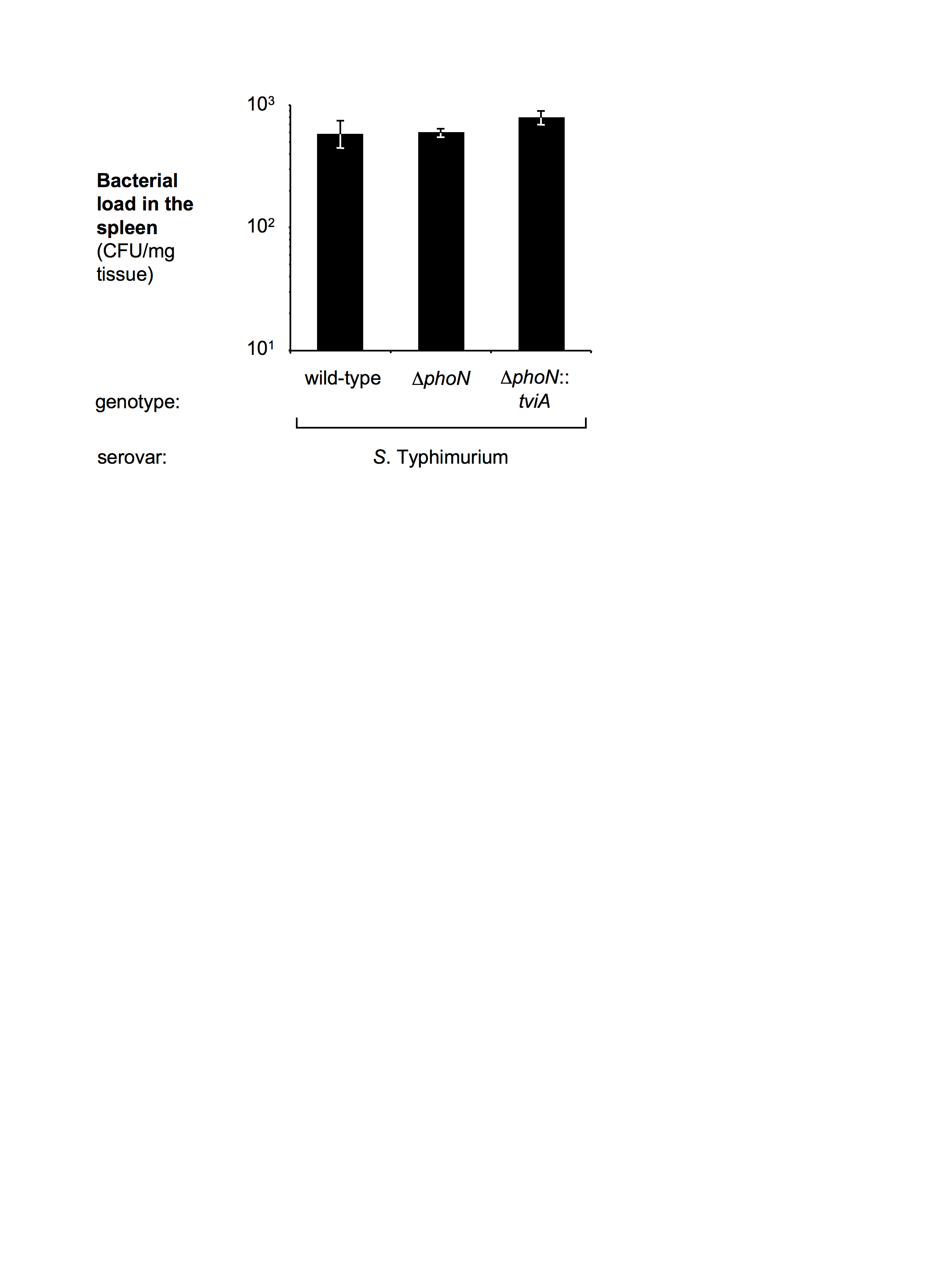

Supplement: Figure S5 — Effect of TviA on growth rate in tissue. Groups of four mice were infected intraperitoneally with the S. Typhimurium wild-type strain (IR715), the ΔphoN mutant (AJB715), and the ΔphoN::tviA mutant (SW474) and the bacterial load in the spleen determined eight hours after infection. Bars represent the geometric mean of three independent experiments ± standard error. (0.17 MB TIF) [file ppat.1001060.s005.tif]
