# Supplementary material for: A Rapid Change in Virulence Gene Expression during the Transition from the Intestinal Lumen into Tissue Promotes Systemic Dissemination of Salmonella
Source: PLoS Pathog. 2010 Aug 19;6(8):e1001060. doi: 10.1371/journal.ppat.1001060 (PMC2924370; doi:10.1371/journal.ppat.1001060)
Supplement: Table S1 — Comparison of TviA-regulated genes in S. Typhi and S. Typhimurium. (0.10 MB PDF) [file ppat.1001060.s006.pdf]

Table S1: Comparison of TviA-regulated genes in *S.* Typhi and *S.* Typhimurium

			S. Typhimurium	*S.* Typhi
			IR715 (pTVIA1) / IR715 (pWSK29)	Δ*tviB-vexE* / Δ*viaB*
Cluster	Annotation in microarray	Gene Symbol	Fold change	Fold change
1	major type 1 subunit fimbrin (pilin)	*fimA*	0.133	0.323
	putative outer membrane lipoprotein	STM1934	0.148	0.341
	flagellar biosynthesis, basal-body outer-membrane L (lipopolysaccharide layer)	*flgH*	0.143	0.385
	flagellar biosynthesis	*fliQ*	0.129	0.400
	flagellar protein FliO	*fliO*	0.124	0.403
	flagellar hook-length control protein	*fliK*	0.136	0.423
	putative Flagellar hook-basal body protein	*fliE*	0.113	0.336
	flagellar biosynthesis, cell-proximal portion of basal-body rod	*flgB*	0.113	0.331
	flagellar protein	*fliJ*	0.110	0.332
	flagellar biosynthesis basal-body MS(membrane and supramembrane)-ring and	*fliF*	0.106	0.345
	flagellar biosynthesis, component of motor switch and	*fliN*	0.109	0.321
	flagellar biosynthesis, component of motor switch and	*fliM*	0.108	0.380
	putative flagella basal body protein	*flgI*	0.105	0.364
	flagellar biosynthesis	*fliL*	0.103	0.385
	flagellar biosynthesis	*flgJ*	0.105	0.311
	flagellar biosynthesis possible export of flagellar proteins	*fliH*	0.102	0.294
	flagellar basal body rod modification protein	*flgD*	0.093	0.339
	flagellar biosynthesis, hook protein	*flgE*	0.105	0.274
	flagellar biosynthesis, cell-proximal portion of basal-body rod	*flgF*	0.094	0.285
	flagellar basal body rod protein	*flgC*	0.089	0.300
	flagellar biosynthesis, cell-distal portion of basal-body rod	*flgG*	0.088	0.379
	flagellum-specific ATP synthase	*fliI*	0.111	0.468
	putative inner membrane protein	*ycgR*	0.079	0.285
	putative cytoplasmic protein	STM3156	0.113	0.236
	hypothetical protein	t1775	0.161	0.274
	putative cytoplasmic protein	STM3155	0.165	0.288
	ssrAB activated gene	*srfB*	0.175	0.294
	putative ATP-dependent RNA helicase-like	STM3154	0.181	0.356

protein			
ssrAB activated gene: predicted coiled-coil structure	*srfC*	0.192	0.343
aerotaxis sensor receptor, senses cellular redox state	*aer*	0.120	0.206
flagellar biosynthesis assembly of basal-body periplasmic P	*flgA*	0.184	0.429
FliZ protein	*fliZ*	0.083	0.225
putative periplasmic protein	*ymdA*	0.201	0.398
ssrAB activated gene	*srfA*	0.195	0.435
putative periplasmic protein	STM1300	0.199	0.257
invasion protein	*invE*	0.157	0.198
invasion genes transcription activator	*hilA*	0.171	0.198
putative AraC-type DNA-binding domain-containing protein	STM4315	0.231	0.274
putative outer membrane protein	STM1328	0.248	0.252
invasion protein	*invA*	0.258	0.360
flagellar protein	*flhE*	0.186	0.592
putative outer membrane or exported	STM1841	0.096	0.630
putative methyl-accepting chemotaxis protein	STM3152	0.122	0.155
flagellar biosynthesis hook-filament junction protein	*flgL*	0.066	0.222
flagella synthesis protein FlgN	*flgN*	0.064	0.207
transcriptional regulator of ftsQAZ gene cluster (LuxR/UhpA	*sdiA*	0.294	0.254
flagellar biosynthesis repressor of class 3a and	*fliS*	0.056	0.244
hypothetical protein	t3059	0.055	0.227
flagellar biosynthesis possible export chaperone for FliD	*fliT*	0.057	0.205
chemotaxis regulator, transmits chemoreceptor signals to flagelllar	*cheY*	0.055	0.192
anti-FliA (anti-sigma) factor also known as RflB	*flgM*	0.050	0.221
flagellar biosynthesis filament capping protein	*fliD*	0.054	0.180
regulator of flagellar biosynthesis, acts on class	*flhD*	0.316	0.318
methyl esterase, response regulator for chemotaxis (cheA	*cheB*	0.046	0.209
flagellar biosynthesis, hook-filament junction protein 1	*flgK*	0.048	0.178
chemotactic response CheY protein phophatase	*cheZ*	0.043	0.198
regulator of flagellar biosynthesis, acts on class	*flhC*	0.301	0.497
cell invasion protein cytoplasmic	*prgI*	0.213	0.128
invasion protein	*invH*	0.356	0.305
transcriptional regulator	*sprB*	0.367	0.324
surface presentation of antigens secretory proteins	*spaP*	0.306	0.161

	putative cytoplasmic protein	*yghW*	0.214	0.790
	purine-binding chemotaxis protein regulation	*cheW*	0.041	0.179
	proton conductor component of motor, torque generator	*motA*	0.039	0.206
	possible AraC-family regulatory protein	*invF*	0.180	0.101
	surface presentation of antigens secretory proteins	*spaO*	0.381	0.188
	cell invasion protein	*prgH*	0.258	0.113
	methyl-accepting transmembrane citrate/phenol chemoreceptor	*tcp*	0.040	0.149
	N-methylation of lysine residues in flagellin	*fliB*	0.086	ND
	putative bacterial regulatory proteins, luxR family	STM4314	0.104	ND
	putative cytoplasmic protein	STM4313	0.166	ND
	30S ribosomal protein S19	*rpsS*	0.176	0.995
	methyl-accepting chemotaxis protein III, ribose and galactose	*trg*	0.089	ND
	putative flagellar biosynthetic protein	*fliR*	0.117	1.065
	putative 50S ribosomal protein	STM1967	0.074	ND
	putative methyl-accepting chemotaxis protein	STM3216	0.234	ND
	putative Diguanylate cyclase/phosphodiesterase domain 3	*yhjH*	0.034	0.396
2	Pathogenicity island encoded protein: homologous to ipgE	*pipC*	0.644	0.075
	cell invasion protein	*sipC*	0.537	0.097
	surface presentation of antigens secretory proteins	*sicA*	0.525	0.107
	cell invasion protein	*sipB*	0.647	0.106
	putative inner membrane protein	STM2870	0.466	0.120
	Salmonella outer protein: homologous to ipgD of	*sopB*	0.387	0.116
	putative flagellar biosynthesis/type III secretory pathway protein	*orgA*	0.423	0.154
	cell invasion protein lipoprotein, may link inner	*prgK*	0.331	0.099
	cell invasion protein	*sipA*	0.550	0.178
	cell invasion protein cytoplasmic	*prgJ*	0.328	0.074
	surface presentation of antigens secretory proteins	*invI*	0.289	0.064
	virulence-associated secretory protein	*spak*	0.243	0.082
	invasion protein outer membrane	*invG*	0.238	0.077
	surface presentation of antigens secretory proteins	*invC*	0.235	0.078
	surface presentation of antigens secretory proteins	*invJ*	0.276	0.060
	flagellar biosynthesis protein	*fliC*	ND	0.089
	pathogenicity island 1 effector protein	*sipD*	ND	0.143

	hypothetical protein	t0349	ND	0.145
	hypothetical protein	t1865	ND	0.148
	putative receptor/regulator protein	t0549	ND	0.148
	methyl-accepting chemotaxis protein II	*cheM*	ND	0.167
	putative cytoplasmic protein	STM2868	0.557	0.250
	regulatory helix-turn-helix proteins, araC family	*hilD*	0.493	0.283
	bacterial regulatory helix-turn-helix proteins, araC family	*hilC*	0.465	0.293
3	flagellar biosynthesis flagellin, filament structural protein	*fliC*	0.014	ND
	Flagellar synthesis: phase 2 flagellin (filament structural	*fljB*	0.056	ND
	putative chemotaxis signal transduction protein	STM2314	0.059	ND
4	invasion-associated secreted protein.	*sopE*	ND	0.030
5	glutamate methyltransferase, response regulator for chemotaxis	*cheR*	0.031	0.177
	methyl-accepting chemotaxis protein I, serine sensor receptor	*tsr*	0.029	0.182
	methyl accepting chemotaxis protein II, aspartate sensor-receptor	*cheM*	0.025	0.174
	flagellar motor protein	*motB*	0.029	0.247
	sensory histitine protein kinase, transduces signal between	*cheA*	0.035	0.131
6	Vi polysaccharide biosynthesis protein	*tviA*	8.174	14.630
7	putative aspartate racemase	STM4510	12.313	1.050
	isoaspartyl dipeptidase	*iadA*	10.505	1.330
	hypothetical protein	t4563	7.295	1.128
	putative transcriptional regulator, LysR family	*yjiE*	7.190	0.947
	putative inner membrane protein	*yjiH*	5.156	1.180
